# Blockade of irradiation-induced autophagosome formation impairs proliferation but does not enhance cell death in HCT-116 human colorectal carcinoma cells

**DOI:** 10.3892/ijo.2012.1329

**Published:** 2012-01-10

**Authors:** ANA CRISTINA DE ALBUQUERQUE-XAVIER, LILIAN GONÇALVES R. BASTOS, JULIO CESAR MADUREIRA DE FREITAS, FERNANDA LEVE, WALDEMIR FERNÁNDEZ DE SOUZA, WALLACE MARTINS DE ARAUJO, JOÃO LUIZ MENDES WANDERLEY, MARCELO NEVES TANAKA, WANDERLEY DE SOUZA, JOSÉ ANDRÉS MORGADO-DÍAZ

**Affiliations:** 1 Division of Cellular Biology, Jose Alencar National Cancer Institute, RJ, Brazil; 2 Division of Experimental Medicine, Jose Alencar National Cancer Institute and Pólo Universitário of Federal University of Rio de Janeiro, RJ, Brazil; 3 Department of Cellular Biology and Parasitology, Carlos Chagas Biophysic Institute, Federal University of Rio de Janeiro, Rio de Janeiro, RJ, Brazil

**Keywords:** autophagy, colorectal cancer, radiotherapy, cell signaling, proliferation

## Abstract

This work was undertaken to gain further information on the molecular mechanisms underlying autophagosome formation and its relation with tumor cell survival in response to radiation in colon cancer. A human colon cancer cell line, HCT-116, was examined with respect to cell survival after blockade of irradiation-induced autophagosome formation by pharmacological interference. Autophagosome formation was confirmed using a kinetic study with incorporated bovine serum albumin gold-conjugate (BSA-Au) analyzed by electron microscopy and an autophagosome-associated LC3B antibody measured by immunofluorescence and Western blotting. Annexin V/PI double staining was used to monitor cell death by apoptosis, and cell cycle profiles by flow cytometry. Ionizing radiation (IR) promoted autophagosome formation in the HCT-116 IR-surviving cells. Pharmacological interference showed that PI3K/Akt and Src were involved in early stages of autophagosome formation. IR alone decreased cell proliferation by arresting cells in the G_2_/M phase, and pharmacological interference of autophagosome formation decreased proliferation, but did not affect cell survival. Also, our data suggest that decreased proliferation caused by PI3K and Src inhibitors could be through S phase cell cycle delay. Our results clearly indicate that blockade of IR-induced autophagosome formation impairs proliferation but does not enhance cell death in colon cancer cells.

## Introduction

Autophagy is a genetically and evolutionarily conserved process that occurs in all eukaryotic cells from yeast to mammals, which is used for the non-selective removal of long-lived proteins, large protein aggregates, and subcellular organelles (1–3). At the beginning of autophagy, portions of the cytoplasm and intra-cellular organelles are sequestered in double membrane-bound structures to form autophagosomes. Subsequently, they fuse with lysosomes to form autophagolysosomes, where the sequestered content is degraded by lysosomal hydrolases or recycled (4).

There is increasing evidence describing the role of autophagy in cancer (5), and various antineoplastic therapies were reported to induce autophagy in human cancer cell lines (6–8). However, fundamental questions regarding whether autophagy kills cancer cells or protects them from unfavorable conditions have not yet been clearly answered (9). Some studies have suggested an inverse relationship between autophagy and malignant growth, as Beclin 1, a key autophagy protein regulator, is a haploinsufficient tumor suppressor gene in mice (10,11) but is frequently absent in human cancers (12). Also, the identification of damage-regulated autophagy modulator (DRAM), a protein necessary to induce p53-dependent autophagy, provides another link between autophagy and tumor suppression (13). Therefore, these studies suggest that defects in autophagy may favor carcinogenesis and that the restoration of autophagy may have promising therapeutic implications in cancer (14). However, there is no specific cancer therapy that targets autophagy in colorectal cancer, consequently a better understanding on the molecular mechanisms by which carcinogens lead to autophagy may contribute to the advancement of a rational clinical therapy in this cancer type.

Autophagy is a multistep process, and various signaling pathways have been implicated in its upregulation and/or downregulation (2,3,15). Nevertheless, independently whether the cell is normal or malignant, the mammalian target of rapamycin (mTOR) serves as the main regulator of autophagy (16). Oncogenic forms of Ras are also implicated in the negative control of autophagy through the activation of class I PI3K (17), and PTEN, the classic negative regulator of PI3K, regulates autophagy suppressing Akt activity leading to autophagy initiation (18). The mitogen-activated kinases (MAPKs) also were reported to regulate autophagy in cancer cells (19). Thus, various signaling pathways may control autophagy, but it is not fully understood if these pathways induce or suppress autophagy formation depending on the cell type. In epithelial tumor cells, such as breast, prostate, and colon cancer cells, it was reported that irradiation induced autophagosome formation, but apoptosis has little or no role in cell death (20–22). However, the cell signaling events underlying the formation of these organelles after IR, particularly in colon cancer cells has been only slightly explored, and it remains unclear if autophagy inhibition contributes to cell death.

Therefore, we hypothesized that IR promotes autophagosome formation in an event mediated by cell signaling pathways activated by this cancer therapy and that autophagy inhibition could lead to cell death. To test this hypothesis, we used the highly invasive human colon cancer HCT-116 cells, which are an appropriate model to investigate the IR-induced effects (23), and blockade of autophagosomes formation by pharmacological interference. Our finding clearly demonstrate that blockade of IR-induced autophagosomes impairs proliferation but do not enhance cell death in colon cancer cells.

## Materials and methods

### Antibodies and reagents

The following antibodies were used in this study: Akt and phospho-Akt antibodies (Cell Signaling Technology, Boston, MA, USA), v-Src and phospho-Src antibodies (Oncogene Research Products, Boston, MA, USA), LC3B antibody (Sigma Chemical Co., St. Louis, MO, USA), α-tubulin antibody (Zymed Lab. Inc., San Francisco, CA, USA). The following secondary antibodies were used: Alexa fluor 488-conjugated antibody (Invitrogen Co., Carlsbad, CA, USA) and horseradish peroxidase-conjugated antibody (Sigma Co.) Both 2-(4-Morpholinyl)-8-phenyl-4H-1-benzopyran-4-1 (LY294002) and 1-tert-Butyl-3-(4-chlorophenyl)-1H-pyrazolo(3,4-d)pyrimidin-4-amine (PP2) were purchased from Calbiochem (La Jolla, CA, USA). Annexin V FITC-conjugated and 3-methyladenine (3-MA) were obtained from Sigma Co., and Zymed Lab., respectively.

### Cell culture, γ irradiation, and pharmacological inhibition

HCT-116 cells (ATCC, number CCL-247, Manassas, VA, USA) were maintained in Dulbelcco’s modified Eagle’s medium (DMEM) containing 10% fetal bovine serum (FBS; Gibco) at 37°C and 5% CO_2_. Irradiation was carried out 48 h after plating (time 0) at 25°C using a 137 Cs Irradiator (IBL 437) with a dose of 8.5 Gy at a dose rate of 2.54 Gy/min. All analyses were carried out 12, 24, 36, 48 and 72 h after irradiation.

For pharmacological interference assays, cells were incubated for 60 min with the kinase inhibitors: 10 μM LY294002 (PI3K) and 170 nM PP2 (Src). Cells were also treated for 60 min with 10 mM 3-MA, a well-known autophagy inhibitor. Cells were rinsed with phosphate buffered saline (PBS), irradiated as described above, and processed for later analysis.

### Annexin V/PI doucble staining

Cells were trypsinized, washed in PBS (pH 7.4), and resuspended in 1X Annexin binding buffer (10 mM HEPES/NaOH, pH 7.4; 140 mM NaCl; 2.5 mM CaCl_2_). A cell suspension with 10^5^ cells (95 μl) was then incubated with 5 μl of Annexin V-FITC for 60 min at 4°C. Prior to flow cytometry analysis, 5 μl of a 20 μg/ml propidium iodide stock solution was added to the cells, and then analyzed with a FACScalibur flow cytometer and analyzed in CellQuest software (BD Bioscience, San Jose, CA, USA).

### Cell cycle analysis

Cells were harvested by trypsinization and washed once with ice-cold PBS. The cells were then stained in the dark with 75 μM propidium iodide and 20 μg/ml RNase A in PBS for at least 30 min in the presence of NP-40. At least 10,000 events for cell cycle were assessed in these experiments using a FACScalibur flow cytometer and CellQuest software.

### Cell proliferation assay

HCT-116 cells (5×10^6^/well) were cultured in 96-well plates and after 48 h of treatment, cell proliferation was assayed in triplicate samples. Cells were fixed in ethanol and stained in a solution of 0.05% violet crystal in 20% ethanol. Then, washed, treated in methanol and the optical densities were measured at 595 nm using a Spectra Max 190 spectrophotometer (Molecular Devices, Sunnyvale, CA, USA).

### Supravital cell staining with acridine orange

Cells were cultured on coverslips for 48 h. After irradiation, acridine orange (Sigma) was added at a final concentration of 1 μg/ml. Where indicated, Bafilomycin A1 (Sigma), a specific inhibitor of the vacuolar H^+^-ATPase, was added to the cells at a final concentration of 0.2 μM before the addition of acridine orange. The coverslips were washed and mounted using n-propyl gallate. The fluorescence was detected using an Axiovert S 100 microscope equipped with a KS300 image analyzer (Carl Zeiss Inc., Germany).

### Immunofluorescence analysis

Cell monolayers were grown on coverslips and subjected to different treatments. Cells were washed, fixed in 4% freshly prepared formaldehyde, and incubated in NH_4_Cl. The cells were then permeabilized with 0.5% Triton X-100 and blocked with 3% BSA in PBS. Subsequently, they were incubated with the primary antibody against LC3B (10 μg/ml) followed by incubation with an Alexa fluor 488-conjugated secondary antibody. The coverslips were washed and then mounted using n-propyl gallate. The cell staining was detected using the Axiovert S 100 microscope.

### Transmission electron microscopy (TEM)

Cells were grown on Transwell polycarbonate filters (0.4-μm-pore size; Costar, Cambridge, MA, USA). After IR, cells were fixed in 2.5% glutaraldehyde, 1% freshly prepared formaldehyde, 0.8% sucrose, and 2 mM CaCl_2_ in 0.1 M cacodylate buffer (pH 7.4). Post-fixation was carried out with 1% osmium tetroxide in cacodylate buffer containing 0.8% potassium ferrocyanide and 5 mM CaCl_2_. Subsequently, cells were dehydrated in a graded series of acetone and embedded in epoxy resin. Ultrathin sections (60 nm) were stained with uranyl acetate and lead citrate, and examined using a Zeiss CEM-900 transmission electron microscope.

In order to monitor autophagosome formation, cells were grown as described above and incubated for 2 h at 37°C with 10 nm BSA-Au. Experiments were also carried out in the presence or absence of protein kinase inhibitors to monitor the involvement of cell signaling pathways in organelle formation, and in the presence or absence of 3-MA. After the treatments, cells were washed, irradiated, and incubated for 12, 36 or 48 h at 37°C. At the indicated time periods, cells were processed for routine electron microscopy as described above. At least 100 cells from each treatment were randomly analyzed.

### Western blot analysis

Total lysates were obtained by homo-genizing cell samples in RIPA buffer (1% NP-40; 0.5% deoxycholate; 0.2% SDS; 150 mM PBS; and 50 mM Tris-HCl, pH 7.4) containing 20 mM sodium fluoride, 1 mM sodium orthovanadate, and a cocktail of protease inhibitors (1:100 dilution). Equal amounts of protein samples were separated by SDS-PAGE and transferred to nitrocellulose sheets. The blots were blocked in TBS-T (20 mM Tris-HCl, pH 7.6; 137 mM NaCl; and 0.1% Tween) containing 1% BSA, incubated overnight with the indicated antibodies, and then visualized using an enhanced chemiluminiscence (ECL) detection kit (Amersham Biosciences, Buckingham, UK). The protein levels of Akt and Src with their respective phosphorylated forms were quantified by densitometry using LabWorks 4.6 software (Bio-Rad). In each case, the specific activity score was calculated using the following equation: Arbitrary score = amount of phosphorylated protein/amount of total protein. The protein levels of LC3B were calculated using α-tubulin as a loading control. The score for non-irradiated cells was normalized as one in each case.

### Statistical analysis

Statistical analysis was performed using two-way ANOVA with a *post hoc* Bonferroni or Dunnet test in three independent experiments. The significantly different values are indicated in the figure legends as P<0.05 or P<0.01. These values are presented as means ± standard deviations (SD) from three independent experiments. Significantly different values relative to the control group, and the values relative to irradiated cells are reported.

## Results

### Effect of IR treatment on the cell viability and apoptosis of HCT-116 cells

**Figure 1:**
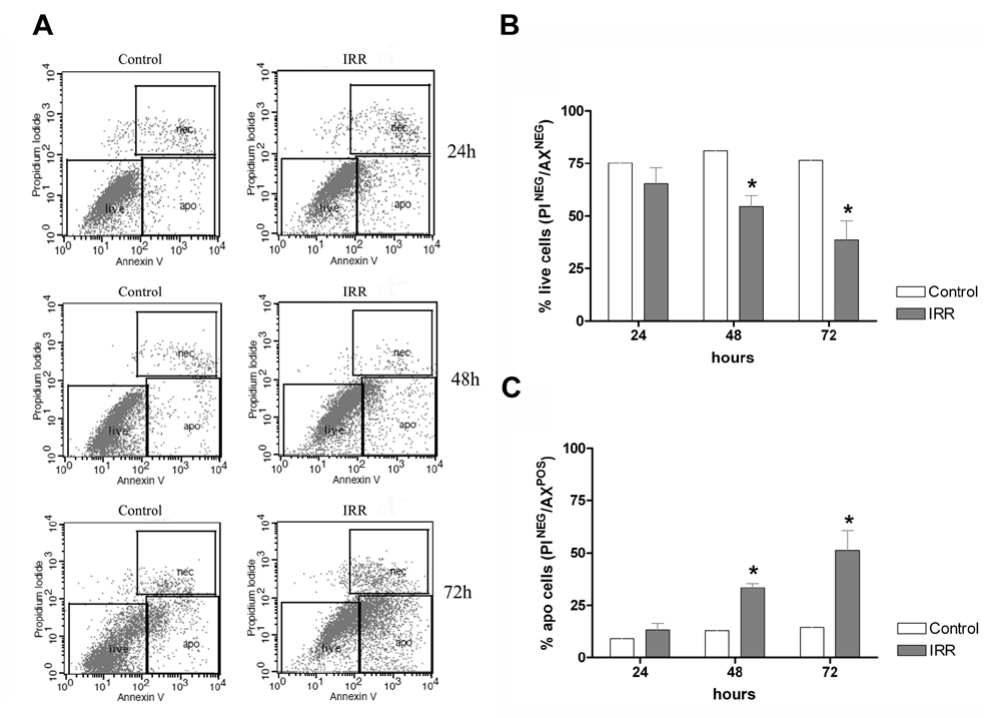
Effect of IR treatment on the cell viability and apoptosis of HCT-116 cells. Cells were grown and irradiated with 8.5 Gy. After 24, 48 and 72 h, the cells were subjected to Annexin V/PI staining and analyzed by flow cytometry (A). Quantitative analysis of PI (B) or Annexin V (C) positive cells. Results are representative of three independent experiments. ^*^Significantly different compared to the control group (^*^P<0.05).

Cells were grown and irradiated with 8.5 Gy. After 24, 48 and 72 h, the cells were subjected to Annexin V/PI staining and analyzed by flow cytometry (Fig. 1A). We observed that treatment of the HCT-116 cells with 8.5 Gy dose after 24 h did not affect the cell survival, but after 48 and 72 h the cell survival was affected in 15 and 40%, respectively, as compared to untreated cells (Fig. 1B). We further examined if IR was able to induce cell death by apoptosis using flow cytometry analysis of cells stained with Annexin V/PI. We observed that IR after 24 h did not alter the levels of apoptotic cells as compared with untreated cells, but after 48 and 72 h a rate of 15 and 60% of apoptotic cells was observed, respectively (Fig. 1C). Based on these results, we used the time of 48 h after IR as treatment condition for all subsequent studies.

### IR promotes acidic vacuole formation that corresponds to autophagosomes

**Figure 2:**
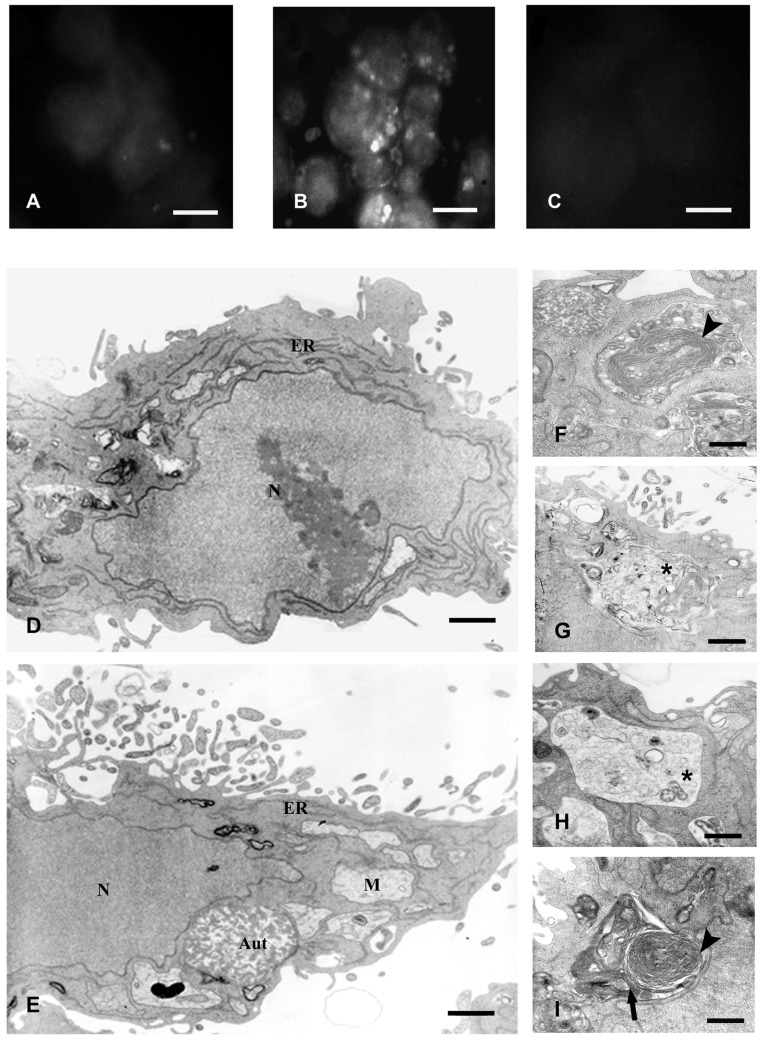
IR promotes acidic vacuole formation that corresponds to autophagosomes. Vital staining with acridine orange of non-irradiated cells (A), 48 h after irradiation with 8.5 Gy (B), and incubated with 200 nM Bafilomycin A1 for 30 min before the addition of acridine orange (C). Bar, 10 μm. (D and E) Representative electron microscopy micrographs of non-irradiated and irradiated cells with 8.5 Gy after 48 h, respectively. The nuclei of irradiated cells exhibited a similar ultrastructure as that observed in control cells and did not show morphological characteristics of apoptosis, such as chromatin margination or nuclear pyknosis. At a higher magnification, it was observed that most of the autophagic vacuoles arise from newly formed lamellar structures (arrowhead) (F) and double-membrane autophagosomes that sequester organelles (arrow) (I) to single-membrane organelles that contain digested material (asterisks) (G and H). (D and E) Bars, 0.2 μm. (F-I) Bars, 0.3 μm. N, nuclei; ER, endoplasmic reticulum; M, mitochondria; and Aut, autophagolysosomes.

We observed that a major population of cell survived to IR after 48 h, while a minor amount was induced to die. Thus, we decided to analyze other events in programmed cell death, such as the additional type II of programmed death or autophagy, which is characterized by the presence of acidic vacuole formation (20). These vacuoles are characterized by labeling with acridine orange, widely known to accumulate in acidic compartments. The majority of untreated cells had only a few labeled vesicles (Fig. 2A); in contrast irradiated cells at 48 h had large fluorescent vacuoles in the cytoplasm (Fig. 2B). We confirm the acidic nature of the vacuoles by incubating the cells with Bafilomicyn A1, a well-known inhibitor of the vacuolar H^+^-ATPase responsible for preventing the proper acidification of lysosomal compartments (24). In the presence of the inhibitor, no acridine orange-labeled vacuoles were observed in irradiated cells (Fig. 2C).

**Figure 3:**
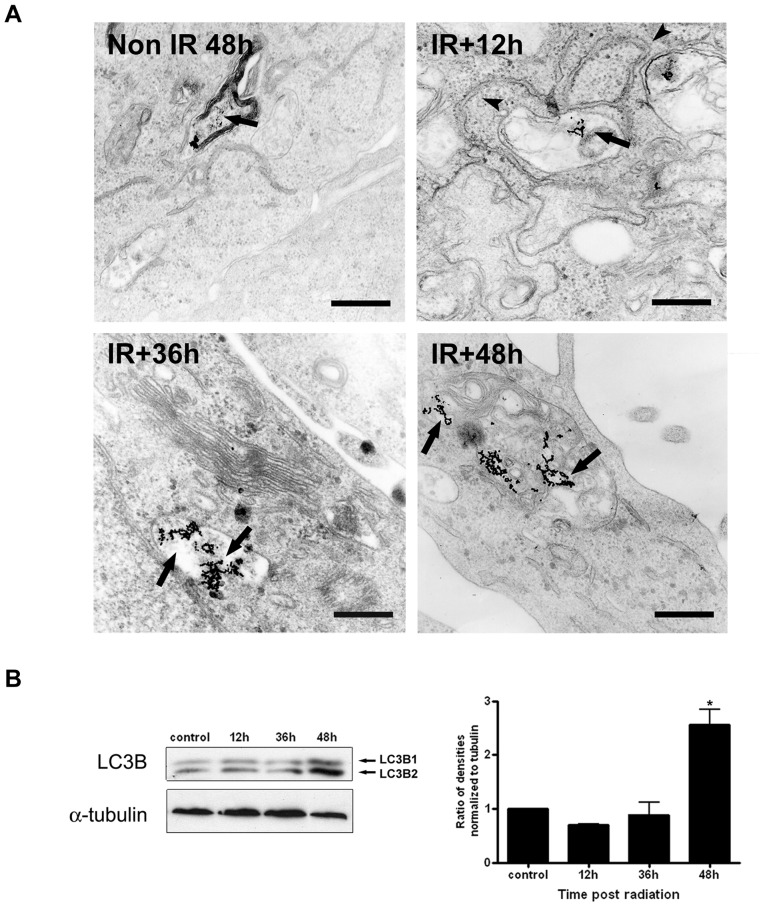
Biogenesis of autophagosomes induced after irradiation. (A) Representative electron micrographs showing a kinetic analysis of the autophagolysosome biogenesis. Cells were first incubated with BSA-Au for 2 h and some cells were irradiated with 8 Gy; all cells were then analyzed by electron microscopy. Control cells had labeling at lysosome-like structures after 48 h of incubation. After 12 h of irradiation, the labeling was present in organelles surrounded by double membranes (arrowheads). Labeling was observed in the autophagosome-like structures after 36 h and within 48 h in mature autophagosomes or autophagolysosomes (arrows represent the BSA-Au marker; bars, 0.3 μm). (B) Western blot analysis of LC3B in control cells and cells irradiated with 8 Gy after 12, 26 and 48 h. Irradiated cells after 48 h displayed a significant increase in both isoforms but primarily in LC3BII. α-tubulin was used as a loading control. Results are representative of three independent experiments. ^*^Significantly different compared to the control group (P<0.05).

Further analysis by TEM showed that the IR-induced acidic vacuoles corresponded to large vacuoles containing electron dense material but the nuclei and organelles had typical morphology similar to control cells and did not show morphological characteristics of apoptosis, such as chromatin margination or nuclear pyknosis (Fig. 2D and E). Forty-eight hours after IR, accumulation of these organelles was observed in ~80% of the analyzed cells, which displayed a core composed of granular, vesicular, or lamellar contents. Furthermore, the organelles were frequently surrounded by smooth and/or rough membrane cisternae or in fusion with smooth vesicles of unknown origin (Fig. 2F-I). Early studies have characterized the integration of the endocytic and autophagic pathways using BSA-Au as a marker of fluid-phase endocytosis (25). Using this approach, we observed the labeling with the marker of lysosome-like organelles in unirradiated cells after 48 h. In irradiated cells the marker was observed in double membranes in apparent sequestration of organelles (12 h), initial autophagosomes contained non-degraded material, indicating that the absence of lysosomal hydrolases (36 h), and in single membrane-bound autophagosomes containing multivesicular material (48 h) (Fig. 3A).

We confirmed these results by Western blot analysis using anti-LC3B, an antibody that recognizes two forms of LC3B (LC3B-I and LC3-II). In Fig. 3B, we show a significant increase in LC3B-II levels 48 h after IR treatment. The increased LC3B-II level is associated with increased autophagosome membranes and correlates with the extent of autophagosomes formation (26).

### Irradiation induces PI3K/Akt and Src activation concomitantly with autophagosome formation at early stages after irradiation

**Figure 4:**
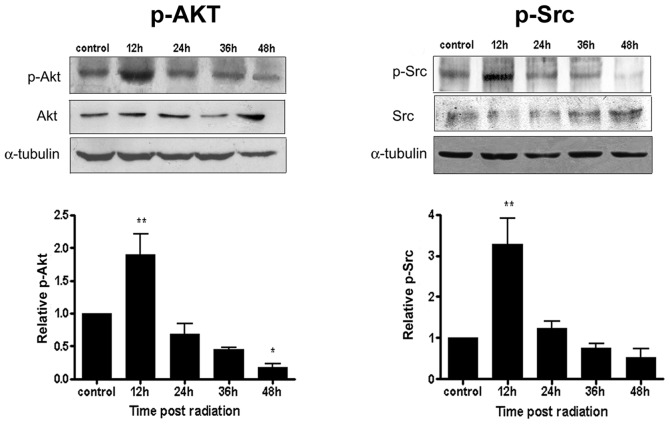
IR induces activation of PI3K/Akt and Src protein kinases. HCT-116 cells were irradiated with 8.5 Gy. Cell extracts were obtained at the indicated times and analyzed for total Akt and Src and their respective phosphorylated forms by Western blotting. A significant increase in the activity of the two kinases after 12 h of irradiation is shown with a progressive decline in their phosphorylation status. α-tubulin was used as a loading control. Results are representative of three independent experiments. ^*^Significantly different compared to the control group (^*^P<0.05); (^**^P<0.01).

To identify cell signaling pathways involved in the formation of autophagosomes, we first analyzed by Western blotting the phosphorylation status of Akt, a downstream target of PI3K, and of Src after irradiation. We observed a significant increase in the phosphorylation levels of these kinases 12 h after IR. At subsequent post-IR times, the phosphorylation of the kinases decreased when compared to control cells (Fig. 4). These data implicate the involvement of PI3K/Akt and Src activation at the initial stages of autophagosome formation, where organelle sequestration and the initial formation of autophagosomes occur.

**Figure 5:**
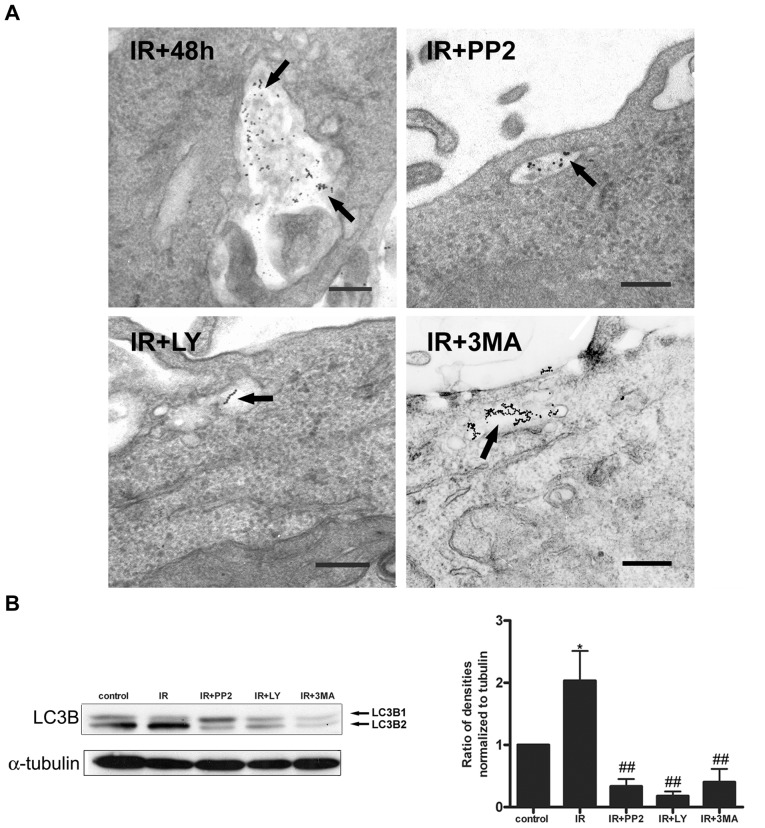
Autophagosome biogenesis is dependent on PI3k/Akt and Src activation in the early stages of irradiation. HCT-116 cells were grown, pretreated with LY294002 and PP2 or with 3-methyladenine for 1 h, and irradiated with 8.5 Gy. After 48 h, the formation of autophagolysosomes was examined by electron microscopy using the BSA-Au marker or by Western blotting with LC3B antibody. (A) An intense BSA-Au labeling inside autophagosomes in irradiated cells and labeled organelles was only observed in early endosomes in the presence of the inhibitors (bar, 0.3 μm). (B) Concomitantly, a significant decrease in the autophagosome marker LC3B was observed after pretreatment with the inhibitors. ^*^Significantly different compared to the control group (^*^P<0.05).

**Figure 6:**
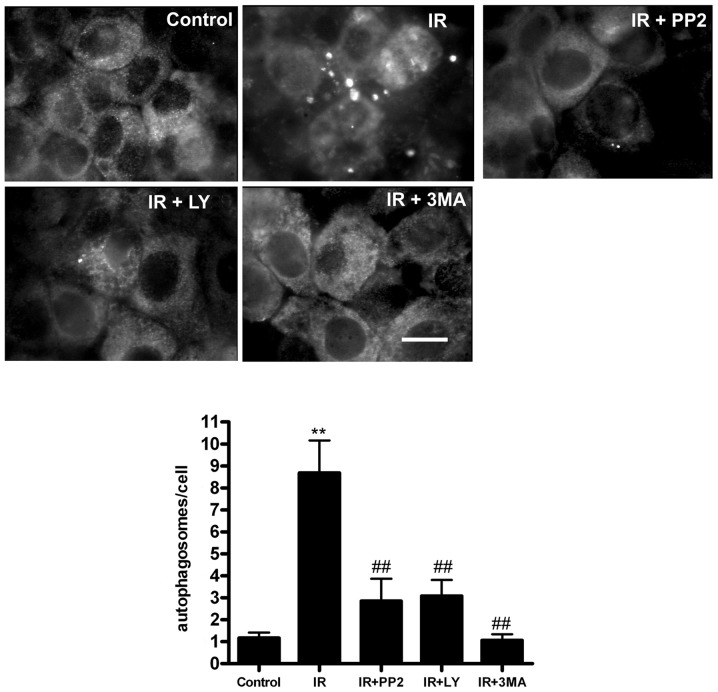
Quantitative immunofluorescence analysis of irradiation-induced autophagosome formation and blockade by inhibitor treatments. HCT-116 cells were grown, pretreated with LY294002 and PP2 or with 3-methyladenine for 1 h, and irradiated with 8.5 Gy. After 48 h, the formation of autophagososomes was analyzed by immunofluorescence using LC3B antibody. The following features were observed: little or no staining in control cells, intense labeling in irradiated cells in the absence of inhibitors, and the disappearance of staining in cells pretreated with inhibitors. Bars, 4 μm. ^**^Significantly different compared to the control group (^*^P<0.01).

We monitored the autophagosome formation in response to PI3K and Src inhibition using the specific inhibitors, LY294002 and PP2, respectively, and 3-MA as a control for autophagy inhibition. TEM analysis showed that ~80% of the irradiated cells displayed a strong BSA-Au labeling into the autophagosomes, and the treatment with the inhibitors caused a blockade of autophagosome formation. Irradiated cells treated with the inhibitors only had organelles resembling early and late endosomes labeled with the marker (Fig. 5A). We further confirmed that the autophagosome formation depends on the early activation of PI3K/Akt and Src by Western blotting using LC3B. Fig. 5B shows that pretreatment with the inhibitors significantly inhibited the expression of LC3B in response to IR. Next, quantitative immunofluorescence using the LC3B marker demonstrated that pretreatment with the inhibitors significantly inhibited autophagosome formation (Fig. 6). Together, these data indicate that the activation of PI3K/Akt and Src during the early stages of irradiation (12 h) is necessary to induce a cell signaling cascade that leads to autophagosome formation in HCT-116 cells.

### Blockade of IR-induced autophagosome formation by PI3K/Akt and Src inhibition impairs proliferation but does not enhance cell death

**Figure 7:**
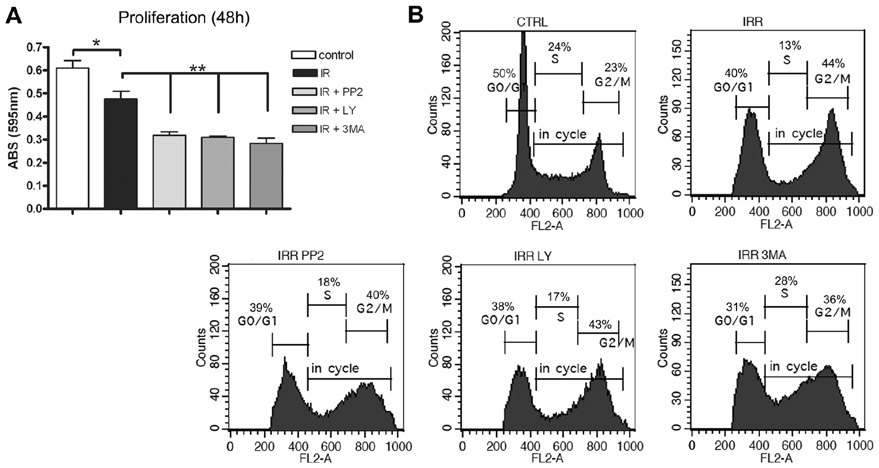
Blockade of IR-induced autophagosome formation by PI3K/Akt and Src inhibition impairs proliferation but do not enhance cell death. HCT-116 cells were grown, pretreated with LY294002 and PP2 or with 3-methyladenine for 1 h, and irradiated with 8.5 Gy. After 48 h, the proliferation (A) and cell cycle analysis (B) were analyzed as described in Materials and methods. Observe that the blockade of IR-induced autophagosomes causes an inhibitory synergistic effect on the total number of irradiated cells at the time of 48 h (A), IR promoted arrest in G_2_/M phase when compared to untreated cells, and the pretreatment with kinase inhibitors and 3MA prior to IR increased the amount of cells in S phase as compared with irradiated cells (B). Data represent the mean ± SD from three independent experiments. ^*^Significantly different compared to the control group (^*^P<0.05).

Various studies suggest that the autophagic response of cancer cells to radiotherapy is a major pathway that may lead to cell death or survival in contrast to apoptosis, which only leads to cell death (27). Autophagy is a temporary survival mechanism under stress conditions, but its inhibition may either promote or inhibit cell death depending on the conditions and agents used (7,28). Thus, we decided to analyze cell proliferation measuring crystal violet incorporation, which correlates with the total cell number, after the blockade of autophagosome formation with PI3K/Akt, Src, and 3-MA. Fig. 7A shows that IR caused a decrease in proliferation after 48 h when compared to non-irradiated cells, and that the pretreatment with the inhibitors significantly decreased proliferation relative to cells treated with IR alone. This suggests that the blockade of IR-induced autophagosomes causes a synergistic inhibitory effect on the total number of irradiated cells at the time of 48 h.

**Figure 8:**
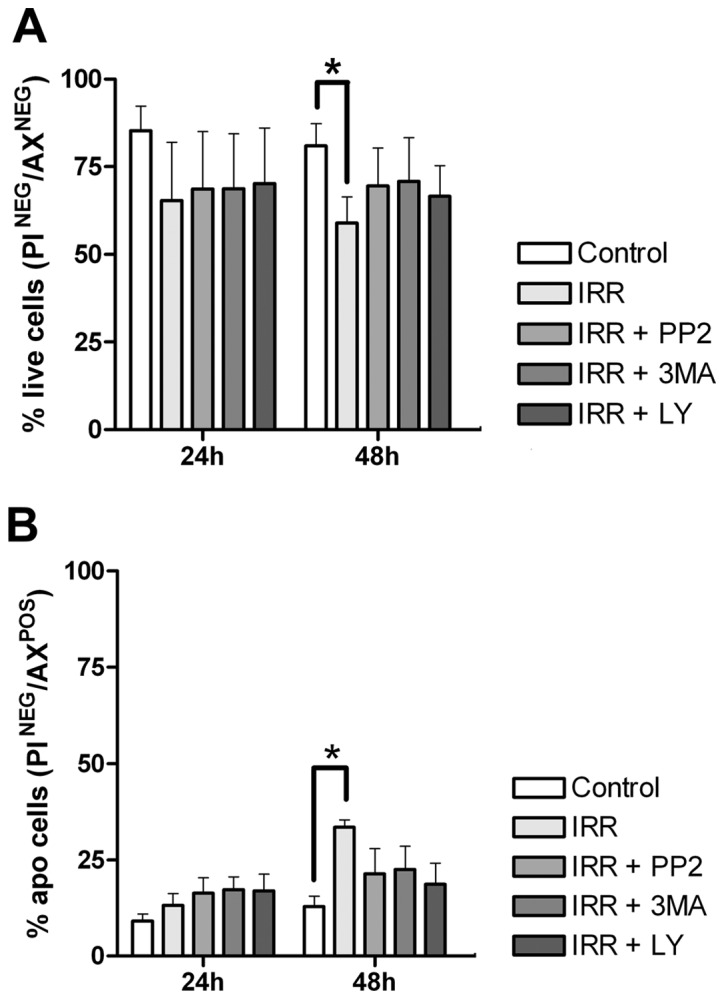
Pretreatment with the inhibitors did not alter the cell viability or the cell death by apoptosis in relation to observed after irradiation. Cells were grown and irradiated with 8.5 Gy. After 24 and 48 h cells were subjected to Annexin V/PI staining and analyzed by flow cytometry. Quantitative analysis of the percentage of PI-(A) or Annexin V-(B) positive cells. Results are representative of three independent experiments. ^*^Significantly different compared to the control group (^*^P<0.05).

In order to determine whether the inhibition of HCT-116 cell growth caused by the blockade of IR-induced autophago-some formation was due to alteration of the cell cycle, we investigated the cycle profiles analyzing the DNA content by flow cytometry. As shown in Fig. 7B, the phases of cell cycle distribution indicated that IR promoted arrest in G_2_/M phase at 48 h, as compared to untreated cells. Pretreatment with kinase inhibitors and 3-MA increased the amount of cells in S phase as compared with irradiated cells. The percentage of pretreated cells in the Sub-G_0_ phase was very similar to those irradiated only (data not shown). At 72 h, the population of cells pretreated with the inhibitors in G_2_/M phase was similar to those irradiated only (data not shown). Taken together, these results suggest that the growth-inhibitory effect as consequence of the blockade of autophagosomes formation could be partly due to its ability to retard S phase of the cell cycle rather than leading cells to apoptosis-mediated death. To investigate in more detail the role of autophagy on the inhibitory effect of proliferation, we examined whether the autophagy inhibitors could attenuate cell survival. We observed that pretreatment with the inhibitors did not alter cell viability (Fig. 8A) or the cell death by apoptosis (Fig. 8B) in relation to those resulting from 48 h after IR. Together, these results indicate that autophagy inhibition decreases cell proliferation but did not enhance the IR-mediated cell death. Additionally, an accentuated effect on these two parameters was observed after 72 h, however none of the inhibitors altered the effects on cell death after IR (data not shown).

## Discussion

Colorectal cancer is the second most prevalent cancer and the third leading cause of cancer deaths worldwide, and radiotherapy remains the major adjuvant therapy to improve survival rates and reduce the risk of local recurrence (29). However, this therapy may contribute to cellular responses related to neoplastic progression. One of these responses involve autophagosome formation as a cell defense mechanism (30), but the precise molecular mechanisms underlying formation of these organelles and its relation with tumor cell survival in response to radiation remain to be defined.

To further address this issue, we initially analyzed whether IR impairs cell viability or induces apoptosis in HCT-116 cells. We observed that IR induced approximately15% of cell death by apoptosis after 48 h of treatment, but 75% of cells survived this treatment. These findings corroborate previous studies showing that irradiated cells develop an adaptive response as a self-defense mechanism that protects cells rather than causing cell death (31). It was shown that depletion or inhibition of p53 could induce autophagy suggesting a key role for the p53 tumor suppressor in the regulation of autophagy (32). However, recently using both WT and p53-null HCT-116 cells that were treated with the cell wall skeleton of *Mycobacterium bovis* Bacillus Calmette-Guerin (BCG/CWS) plus IR, it was shown that p53 was not involved in autophagy induction (33). Therefore, these findings also indicate that autophagy and apoptosis use different mechanisms of control.

Various studies have suggested that anticancer therapies, such as hormonal agents, chemotherapy and IR, frequently induce autophagy, in most cases as a pro-survival response potentially contributing to treatment resistance (34,35). Thus, we evaluated autophagosome formation, which is the most distinctive feature of autophagy. Our results showed that IR induced the accumulation of large vacuoles derived from the autophagic pathway, as characterized by the following parameters: a) acidic nature, b) enclosure by a double membrane, c) presence of degraded material derived from organelles such as the endoplasmic reticulum or mitochondria in their lumen, and d) labeling with LC3B (Figs. 2 and 3). As previously reported, these criteria are well-known characteristics of autophagosome formation (36).

Previous morphological studies have shown a link between the endocytic and autophagic pathways (37); therefore we used the convergence of these pathways to monitor the formation of IR-induced autophagosomes using BSA-Au, a classic fluid-phase endocytosis marker, and TEM analysis (25). Using this approach we showed that IR induced the formation of autophagosomes after 48 h (Fig. 3A), which was confirmed by measuring LC3 expression levels by Western blotting (Fig. 3B). There are no previous reports demonstrating the biogenesis of IR-induced autophagosomes in invasive colon cancer cells. The present study reinforces a previous report suggesting that the integration of autophagic vacuoles with vacuoles of the endocytic pathway is a prerequisite of autophagosome maturation (1), and provide a valuable model to investigate molecular mechanisms that control autophagosome biogenesis.

Autophagy is a dynamic process regulated by several cell signaling pathways (5,38), however, the molecular control of IR-induced autophagy, particularly in colon cancer cells, remains poorly understood. In human breast tumor cells, a link between autophagic responses to IR and mTOR signaling was reported (31. It was shown that clinically relevant IR doses increased the phosphorylated forms of mTOR, Akt, and S6 ribosomal proteins (39). Therefore, inhibitors of the PI3K/Akt/mTOR pathway have emerged as important and attractive therapeutic strategies for cancer therapy. Our results demonstrated that PI3K/Akt and Src signaling were involved in autophagy control (Figs. 4-6). We suggest that IR, known to activate growth-factor receptors (40,41), could increase the activity of Ras family oncoproteins causing subsequent activation of downstream effectors such as PI3K/Akt and Src, which in turn, may modulate autophagosome formation. Once these IR-activated signaling pathways are correlated with cell survival, we can also speculate that IR-induced autophagy in this cell type is an attempt to avoid apoptosis in response to this treatment. It is important to point out that LY294002 is a known inhibitor of autophagy as well as of PI3K signaling (42) and suppresses the activity of the downstream Akt. However, this inhibitor also induces rather than inhibits autophagy in malignant glioma cells. One explication to this controversy could be that the effects of LY294002 on autophagy depend on the cell type or treatment conditions, such as concentration used and exposure time, which can affect the mTOR pathway (43).

We further analyzed cell proliferation and survival rates to investigate if the blockade of autophagy could be sufficient to enhance cell death induced by IR. Interestingly, the concentration of inhibitors that induced autophagy blockade did not increase apoptotic cell death but significantly inhibited the proliferation of irradiated cells (Figs. 7 and 8). This latter observation could indicate that the decreased proliferation induced by the blockade of autophagy was due to a delay of the cell cycle in S phase. To confirm this hypothesis we analyzed the cell cycle progression 72 h after IR, and observed a restoration of the cell population in G_2_/M in the group treated with kinase inhibitors as compared with the irradiated group only. This suggests that the presence of autophagosomes is important to progress the cell cycle from S to G_2_/M where G_2_ checkpoint allows the cell to repair the DNA damage after IR. Recently, it was shown that IR-induced autophagy promotes post-IR cell survival and contributes to cellular radioresistance in breast cancer cell lines (44); however experimental evidence to explain this conclusion was not explored. Of note, the PI3K/Akt pathway is a survival pathway that via its inhibition does not always cause substantial apoptosis, therefore, additional treatment with other agents is necessary to cause abnormal autophagosome accumulation and leads to accelerated cell death (45).

In conclusion, we showed that IR-induced autophagy is a initial mechanism of tumor cell survival, and that autophagy inhibition induces a delay of the cell cycle in S phase rather than leading cells to apoptosis-mediated death. Therefore, cell death in response to autophagy inhibition may vary among each type of cancer due to the different biological characteristics and therapeutic treatments, as previously reported (46). In the future, the understanding of the molecular mechanisms underlying IR-induced autophagy as well as the combination with other drugs that interfere with other defense programs may increase the efficacy in radiation oncology.
